# The Tribulations of a Medical Student

**Published:** 1887-09

**Authors:** 


### THE TRIBULATIONS OF A MEDICAL STUDENT.

By "QUIGLEY," M. D.

MR. EDITOR—You ask me to write you a reminiscence of my student days. Now, I don't like reminiscences. Somehow my Thaumaturgic spade always digs up dry bones, and the particular one you want—well, I would as soon write you a ghost story as I sit here alone in the office, to-night as to recall this special reminiscence. There are some things we never can forget; but, like Banquo's ghost, they will not keep down, but rise again.

I was young then, and am getting old now. The frosts of many winters are whitening my locks. I have seen many of the mile-stones of time flit by me. I have learned and forgotten many things; but this ghost of my student days still looms behind me like the spectre of the "Brocken." It was my first "granny" case.

I was a student in medicine, had attended my first course of lectures, and was spending my inter-collegiate vacation as "Interne" at the old Tennessee State Hospital. Old Dr. D——— called on me one day, and informed me that he had been engaged to attend a Mrs. H———, who lived in the neighborhood, in her confinement, then about two weeks off; that he was compelled to be absent, and he wanted me to take charge of the case. I could not refuse, and agreed to do so. The next day the old doctor brought me over a set of harness to put on her when in labor, and explained their application. There were feet rests, hand pulls, belly-bands, shoulder braces, and the Lord only knows what else. I only remember I was afraid to try and make the application, and gave it up in disgust.

Two weeks—well, if there ever were two long weeks, they were the ones. I was in a constant state of dread. The call of the angel Gabriel and that of Josh H——— were all the same to me. I would have as soon heard the one as the other.

Finally, one morning, as the sun rose, I had to rise, too, at the call of Josh H———. I slipped into a new broadcloth coat which had cost me forty-five dollars, and repaired to the cottage. Shortly after my arrival, I requested an examination, which was readily accorded. Now, the *tactus eruditus* in my case had never been developed—in fact, there was no *eruditus* at all; but I assured the family all would be right, which seemed to impart a great deal of consolation. I was not, however, so happy. I had felt the immense size of the abdomen, and, for the life of me, I could not see how there could ever be an adaptation of the means to the *end.* I remembered, however, that my old professor, Dr. Watson, had explained the wonderful resources of nature, and I had faith, because perforce I was compelled to; but my faith was in Dr. Watson's words, and not based upon the substance of things unseen by me. I mentally cogitated: "Now, old dame, if you come out all right in this thing—the top dog in the fight—you will have to be a hustler and do about, for you can't get any assistance from me, but I never will doubt your vast powers and conservatism; no, never."

The pains were slight, and, after a few hours, some hemorrhage took place; but I remembered Professor W——— had spoken to us about a *show.* I remained satisfied for a few hours; but as it turned out, it was not only a *show* to me, but a whole circus and a hippodrome thrown in. Telling the family that I was compelled to be absent for a short time, but that I would return in time, I made a bee-line for the office of Professor W———, and consulted him. He kindly told me that as soon as the head engaged in the pelvis it would probably cease; and I returned to my charge.

Hours passed, and still but little change. A young girl, one of the burgeoise, who did not seem to appreciate that she was at a questionable place of propriety, had accompanied her mother, and was munching apples under an apple tree in the yard. Every few minutes I would see my patient, and then rejoin the girl, and in my desperation make love. (But I must confess I began to have, like the good old Vicar of Wakefield did for his filly, a most hearty contempt for the sex). Well, Mr. Editor, in my desperation I ate apples until I felt like a cider press, and had almost as much *embonpoint* as my patient, and wished I was a little girl baby in my mother's arms instead of a doctor.

About 11 o'clock at night I took another observation. The waters had not broken, but, O horror of horrors, there was something, but I could not tell what. My manipulations ruptured the membranes, but still I could not make out the presentation. I felt a something, but what could it be? It was not a foot or a hand, because there were no toes or fingers. It was not a scrotum or a tongue; but what could it be? (It was an ear). I could not tell. Unaccountably to me, the pains ceased, and I sat down in utter despair, with no hope but in the conservatism of nature. The old crones around me became uneasy, and commenced to debate the situation.

"Go out, Sallie, and chunk that dog out from under the house. It is a bad sign to hear a dog howl; it is almost a sure sign of death. I remember poor Suckey More died arter a dog howled."

"Yes," said another, "and did you hear that screech owl in the apple tree awhile ago? It is almost as good a sign as fur a dog to howl."

"Pore Miss ———," said another. "Pore critter, she hes suffered powerful to-day. She hes been jest like pore Nancy Sweet the day she dide, but maybe the Lord will pervide."

Now, Mr. Editor, in my helplessness I almost wished a dozen screech owls and dogs would make a call for me. I was nervous. I felt that I was a galvanic battery, and every hair on my head stood straight out, and was an electrode. My hands felt cold and clammy, like Uriah Heep's. I placed them on my brow; it was bathed in perspiration, and felt like a wet tombstone. I could stand no more, and requested that Dr. McE——— be called in consultation. He came, recognized the presentation, very kindly corrected it, and telling the family that I had done right and all that could have been done, that all would soon be right, only give nature a chance, took his departure after reassuring me. Poor fellow, he has long since crossed over where doctors most do congregate, but I shall always love his memory, and feel willing to mend harp strings for him on the other side.

To make a long story short, just before day the baby was born. The old women had prepared a tub half full of water in the middle of the room, and insisted that the doctor always had to wash the first baby he grannied. Not wishing to seem ignorant of the literature of the profession and a custom which had come down to us through the centuries ever since the perennial glories of Eden first tinted the world, when Abel was born to Adam (Dr. Bowling said Adam was a doctor, for Eden could not have been perfect without one), I larded and egged the baby well and essayed my task. The first motion I made to seat it in the tub, it slipped from me. I caught it up, and it slipped in again. Everything was now in an uproar. Babel or a grand symphony from a ten-acre field of jackasses could not have equalled it. "Take it out," "It will drown," "It will scald to death," "Pore thing, it is almost gone now," were some of the consoling remarks I heard. I still had my new coat on. In my desperation I formed a cradle of my arms, and scooped the baby out. The old women now took charge of it, and I retired to another room to wash my coat sleeves. But the devil seemed to have been turned loose in that house, and I found the cleansing operation utterly impracticable, and as difficult as the grannying of the baby.

But my trouble was not over. "Run here quick, doctor, the baby's blue and got a fit." I readily recognized a case of morbus ceruleus, and I remembered Professor Watson had said turn it on one of its sides, but I could not remember which. I turned it on its left side, and it bounced around the bed like pop-corn in a hot skillet. I turned it upon its right side, and the spasm subsided.

Well, Mr. Editor, that baby died, and I—well, by a fortuitous concatenation of heterogeneous circumstances I still survive, and you can see upon my sign:

DR. QUIGLEY.

OBSTETRICAL AND GYNÆCOLOGICAL

CALLS NOT ATTENDED.

